# Public Perception of Artificial Intelligence in Medical Care: Content Analysis of Social Media

**DOI:** 10.2196/16649

**Published:** 2020-07-13

**Authors:** Shuqing Gao, Lingnan He, Yue Chen, Dan Li, Kaisheng Lai

**Affiliations:** 1 Faculty of Psychology Beijing Normal University Beijing China; 2 School of Communication and Design Sun Yat-Sen University Guangzhou China; 3 Guangdong Key Laboratory for Big Data Analysis and Simulation of Public Opinion Guangzhou China; 4 School of Journalism and Communication Jinan University Guangzhou China

**Keywords:** artificial intelligence, public perception, social media, content analysis, medical care

## Abstract

**Background:**

High-quality medical resources are in high demand worldwide, and the application of artificial intelligence (AI) in medical care may help alleviate the crisis related to this shortage. The development of the medical AI industry depends to a certain extent on whether industry experts have a comprehensive understanding of the public’s views on medical AI. Currently, the opinions of the general public on this matter remain unclear.

**Objective:**

The purpose of this study is to explore the public perception of AI in medical care through a content analysis of social media data, including specific topics that the public is concerned about; public attitudes toward AI in medical care and the reasons for them; and public opinion on whether AI can replace human doctors.

**Methods:**

Through an application programming interface, we collected a data set from the Sina Weibo platform comprising more than 16 million users throughout China by crawling all public posts from January to December 2017. Based on this data set, we identified 2315 posts related to AI in medical care and classified them through content analysis.

**Results:**

Among the 2315 identified posts, we found three types of AI topics discussed on the platform: (1) technology and application (n=987, 42.63%), (2) industry development (n=706, 30.50%), and (3) impact on society (n=622, 26.87%). Out of 956 posts where public attitudes were expressed, 59.4% (n=568), 34.4% (n=329), and 6.2% (n=59) of the posts expressed positive, neutral, and negative attitudes, respectively. The immaturity of AI technology (27/59, 46%) and a distrust of related companies (n=15, 25%) were the two main reasons for the negative attitudes. Across 200 posts that mentioned public attitudes toward replacing human doctors with AI, 47.5% (n=95) and 32.5% (n=65) of the posts expressed that AI would completely or partially replace human doctors, respectively. In comparison, 20.0% (n=40) of the posts expressed that AI would not replace human doctors.

**Conclusions:**

Our findings indicate that people are most concerned about AI technology and applications. Generally, the majority of people held positive attitudes and believed that AI doctors would completely or partially replace human ones. Compared with previous studies on medical doctors, the general public has a more positive attitude toward medical AI. Lack of trust in AI and the absence of the humanistic care factor are essential reasons why some people still have a negative attitude toward medical AI. We suggest that practitioners may need to pay more attention to promoting the credibility of technology companies and meeting patients’ emotional needs instead of focusing merely on technical issues.

## Introduction

### Background

High-quality medical resources are in great demand worldwide. The World Health Organization estimates that there is a global shortage of about 4.3 million doctors and nurses, a problem that poses a more serious threat in developing countries [1]. The application of artificial intelligence (AI), a field of computer science that aims to mimic human cognitive functions with computer algorithms [2,3], in medical care can reduce the burden on doctors and nurses, improve patient care, and alleviate the human resource crisis in health care [4]. In recent years, AI has made breakthroughs and has begun to be widely used in various areas of daily life, such as autonomous driving [5], weather forecasting [6], and health care practices [7].

With the increasing availability of medical data and the development of algorithms, AI has become an evolving trend in the medical field [8,9]. In medical diagnosis, AI can improve accuracy through image recognition technology and semantic analysis. In 2017, a Stanford University study revealed that AI beat human doctors in skin cancer diagnosis by attaining more than 90% diagnostic accuracy [10]. In the context of medical decision making and treatment, with the help of increasingly sophisticated algorithms and delicate instruments, AI technology can provide high-quality therapeutic options and perform specific operations. For example, the first robotic hand, developed by the research team of the American Children’s National Health System, could handle soft tissue automatically [11]. In health management, researchers from the Las Vegas Department of Health have applied machine learning to Twitter data and developed an AI system that can help prevent foodborne illnesses [12]. In preventive health care, increasingly rich medical and health data provide opportunities for more accurate health monitoring and disease prevention. Recently, through a deep learning approach, researchers from DeepMind developed a model that was able to predict acute kidney injuries 48 hours in advance [13].

Although medical AI has gone through many technical advances, its usage and social influence (eg, whether AI doctors would replace human doctors) have attracted the attention of the government, media, and society. Existing research mainly focuses on revealing the attitudes and views of AI experts or medical professionals toward medical AI. Previous studies indicated that the overwhelming majority of technical experts in the field of biomedical informatics believe that AI will revolutionize many medical fields [14]. Other experts even predict that human doctors are at risk of being replaced by AI as it gets closer to general human intelligence [15]. Medical professionals, including doctors and medical students, admit that AI can outperform human doctors in some areas. However, they also considered the potential of AI to be limited and are not worried about being replaced by AI [16-18]. However, it is not known how medical AI is perceived by the general public, and few studies have empirically explored the public view on medical AI.

Compared to AI experts and medical professionals, the general public has less subject knowledge, but they are the ultimate users of medical AI. The public’s attitude toward AI in health care is crucial. Public perception and attitudes toward AI in medical care may affect the development progress of AI products (eg, collecting sufficient data from the public for machine learning) in the early stage. In contrast, public acceptance of AI products may exert an influence in the middle and late stages. Therefore, we suggest that the healthy development of the medical AI industry depends, to a certain extent, on whether practitioners in related fields comprehensively understand the public’s views on AI in medical care.

The development of information science and the popularization of social media have made it possible to study the psychology and behavior of a large population based on the amount of behavioral data available from platforms such as Twitter and Facebook [19]. Compared with the questionnaire survey and experimental research, social media analysis in general has higher ecological validity and can reflect the opinions of large groups of people more objectively [20]. In recent years, researchers have started to use social media to study public awareness about certain illnesses, such as lung cancer [21] and cardiovascular disease [22]. It has also been used to explore public perceptions about health care (eg, cardiopulmonary resuscitation [23], vaccines [24]), and the application of new technologies in disease treatment (eg, virtual reality [25]).

Sina Weibo (Sina Corp), which is similar to Twitter, is one of the most popular social media platforms in China and boasts approximately 500 million daily active users from around China [26,27]. A growing body of literature has identified Sina Weibo as a useful platform for public health research, and its data have been used for studies on medical topics such as cancer misinformation [28], depression-related discourses [29], and organ donation awareness [30]. With the rapid development of medical AI, it has become an increasingly important topic of concern for the media and public. Over time, more posts and discussions about people’s feelings, opinions, and concerns regarding medical AI have emerged on social media platforms, including Sina Weibo. This provides an excellent opportunity to study the general public’s perception of AI in medical care based on social media posts.

### Objectives

To the best of our knowledge, no study has comprehensively examined the views of the general public on AI in medical care. To address this gap, we explored the public perception of medical AI on social media through a content analysis of a large amount of data generated from the largest social media platform, Sina Weibo. Specifically, we focused on the following three questions: (1) What are the main medical AI–related topics about which the public is concerned? (2) What are the attitudes of the public toward AI in medical care? (3) Do people believe that medical AI can replace human doctors?

## Methods

The data used in this study were collected from Sina Weibo through its application programming interface. First, we established a data set comprising 16 million Weibo users throughout China, crawled these users’ public posts from January 1 to December 31, 2017, and collected their public registration information (including gender, age, and geographic location). Second, we identified 4515 posts from this data set that contained at least one AI-related keyword (eg, “AI” or “artificial intelligence”) and one medicine-related keyword (eg, “medicine,” “treatment,” or “health”). Third, we cleaned these posts further by manually inspecting and excluding 2000 invalid posts. These invalid texts were mainly caused by the keyword “AI,” as it is usually used as the phonetic transcription of “爱,” which means “love” in Chinese. Moreover, we also excluded invalid posts in which “AI” referred to Adobe Illustrator. Finally, we obtained 2315 posts for further analysis.

Based on these 2315 posts, we first outlined the general characteristics of public attention toward AI in medical care. Specifically, we examined the dynamic fluctuations of public attention over time and revealed the user profiles (such as gender, age, and region) of people who were concerned about medical AI through chi-square tests at the user account level. We then focused on public interest in and attitudes toward medical AI through content analysis. Referring to the coding methods used in previous research [30], we used a direct content analysis approach to code these posts, including the following: (1) topics of public concern regarding AI in medical care, (2) public attitudes toward AI in medical care and the reasons for them, and (3) public opinion on whether AI can replace human doctors. For example, for the thematic content analysis, two researchers screened the posts while one of them proposed a codebook for topic categories. A different researcher then evaluated the categories and discussed the framework with the first two researchers. Thereafter, the two researchers discussed and resolved the disparities in coding and reached a consensus on the classification of all topics. Subsequently, they coded approximately 10% (n=236) of posts to verify intercoder reliability. The κ score for the thematic content analysis was 0.82, which was acceptable. Finally, they independently completed the coding of the remaining posts. In this process, posts that contained more than one topic category were categorized as the more relevant of the options. The coding process of public attitudes toward AI in medical care and public opinion on whether AI can replace human doctors was consistent with the processing of the topics of public concern regarding AI. Furthermore, their intercoder reliability was also acceptable (κ scores were 0.80 for public attitudes toward AI in medical care and 0.92 for public opinion on whether AI can replace human doctors).

## Results

### Public Attention Toward AI in Medical Care

**Figure 1:**
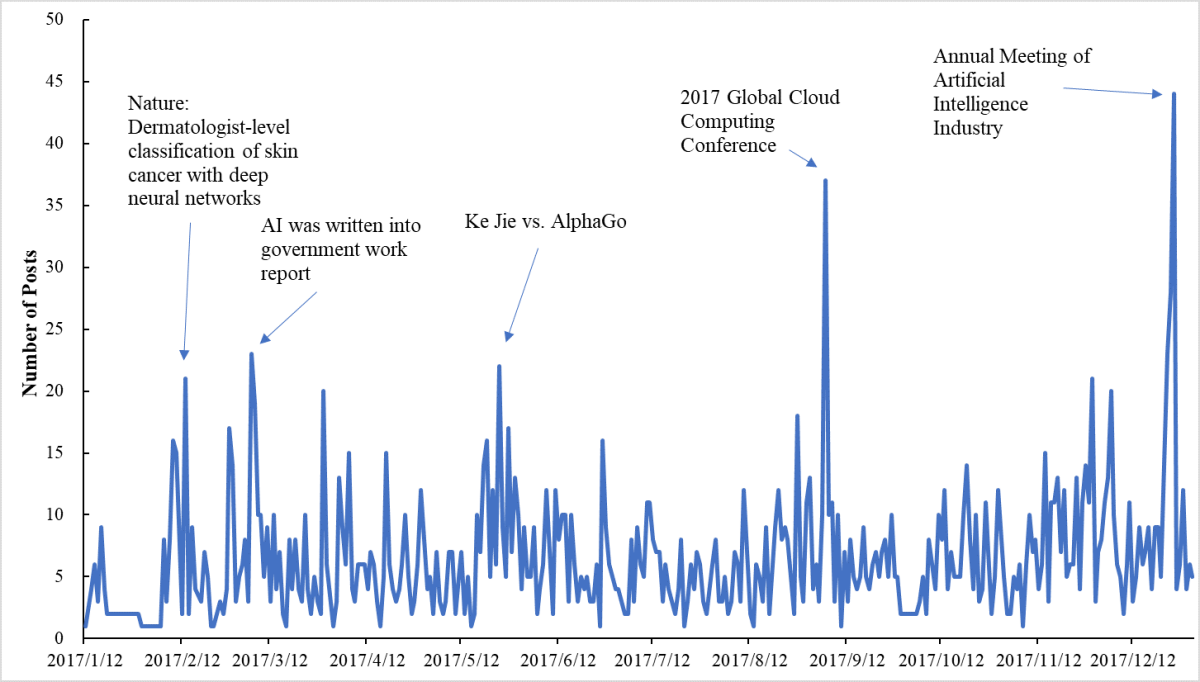
Number of posts about artificial intelligence in medical care from January to December 2017.

To explore the characteristics of public attention toward medical AI, we first examined the dynamic fluctuation in people’s discussions of AI in medical care over time. Figure 1 shows the daily counts of all posts related to medical AI from January to December 2017. Public attention toward AI in medical care demonstrated obvious event-driven characteristics. Whenever a widely reported event related to AI occurred, public attention surged accordingly. We found that there were three main categories of events that could drive public attention toward AI in medical care. The first category comprised messages about improvements and breakthroughs in AI technology, such as when AI beat human doctors at diagnosing skin cancer [31]. The second category comprised information or regulations related to AI released by the government or official institutions, such as when AI was written into a government work report and the 2017 Global Cloud Computing Conference was held. The third category comprised social entertainment events related to AI, such as AlphaGo AI defeating Ke Jie, the world’s number one Go player.

In addition to the temporal characteristics, we explored users’ profile characteristics to identify those who were most interested in medical AI. However, since not every user had publicly disclosed their account information, we limited our analysis to those who had. Of these 1764 accounts, 1294 (73.36%) were attributed to male users and 470 (26.42%) were female users (Table 1). Males paid more attention to AI-related medical topics than did females, even after controlling for the gender ratio of the 16 million users in the data set (χ^2^_1_=948.5; *P*<.001). In terms of age, users under 30 years old paid less attention to AI medical topics than did users over 30 years old, and this was significantly lower than the proportion of young to old users in the data set (χ^2^_1_=491.2; *P*<.001). Moreover, users from the regions (provinces) with above-average incomes showed more interest in medical AI than did users from regions with below-average incomes, at a level significantly higher than the proportion of such users in the data set (χ^2^_1_=90.7; *P*<.001).

**Table 1:** Overview of the general demographics of the users who were interested in medical artificial intelligence.^a^

General demographics		Users in analysis, n (%)	Users of data set, n (%)
**Gender**			
	Male	1294 (73.36)	6,267,548 (37.80)
	Female	470 (26.42)	10,311,693 (62.20)
**Age (years)**			
	≤30	231 (47.53)	6,297,207 (84.22)
	>30	255 (52.47)	1,180,058 (15.78)
**Region**			
	Above-average income	1128 (74.60)	7,858,752 (62.76)
	Below-average income	384 (25.40)	4,662,573 (37.23)

^a^Our statistics were limited to those users who disclosed their demographic information on the corresponding indicators publicly, so the actual number of users on different demographic indicators may be different.

### Thematic Content Analysis

We employed thematic content analysis to explore the topics of public discussion on medical AI. The results showed that such contents fall into three major categories, including 10 subcategories (Table 2).

The first major category, “Technology and application” (n=987, 42.63%), focuses mainly on what AI is and which medical fields it can be applied to; people discussed technical issues (n=109, 4.71%) related to AI technology, as well as its general uses (n=210, 9.07%) and specific uses (n=668, 28.85%). With regard to the specific uses of AI, its technological application in the medical field was the most popular topic; people showed the most interest in the application of AI in diagnosis (n=436, 18.83%) and treatment (n=146, 6.31%), and some interest in prevention (n=52, 2.25%) and recovery (n=34, 1.47%).

The second major category, “Industry development” (n=706, 30.50%), is mainly concerned with the development and trends of medical AI and related industries. People were particularly concerned with the development of related companies (n=331, 14.30%) such as Baidu, Alibaba, and Tencent. They also discussed investment and market (n=186, 8.03%) and industry expectations (n=142, 6.13%), in which they estimated the financial performance and investment possibilities in such an industry. However, few mentioned policy and law (n=47, 2.03%) in their posts. Given that medical AI is new, there are limited regulations and laws related to it for people to discuss.

**Table 2:** Topics of public concern regarding artificial intelligence in medical care (N=2315).

Topics				Posts, n (%)		Definition		Example post
**Technology and application**				987 (42.63)		What is AI^a^ and in which medical fields can it be applied?		N/A^b^
	Technical issues		109 (4.71)		Technical discussions on AI in medical care		“Massive medical data is indispensable for AI medical treatment”	
	General uses		210 (9.07)		General public discussions on AI in medical care without pointing out specific medical areas		“I am looking forward to AI being applied in medicine, autonomous vehicles, and other fields so that ultimately we will all be beneficiaries of AI”	
	**Specific uses**		668 (28.85)		Medical AI applied to specific medical fields		“Vigorous development of the application of AI in treatment and rehabilitation.”	
		Prevention	52 (2.25)		Application of AI in medical prevention		“How amazing AI is to be able to predict Alzheimer’s nine years in advance.”	
		Diagnosis	436 (18.83)		Application of AI in medical diagnosis		“Google’s deep learning AI can diagnose cancer with an accuracy rate of 89%, while the accuracy rate of human diagnosis is currently 73%.”	
		Treatment	146 (6.31)		Application of AI in medical treatment		“Intelligent doctors can list multiple treatment plans in 10 seconds”	
		Recovery	34 (1.47)		Application of AI in medical recovery		“Intelligent family service robots have achieved mass production and application; robots helping in the recovery and assistance of the elderly and disabled are at the prototype production stage.”	
**Industry development**				706 (30.50)		Public attention toward and views on the medical AI industry		N/A
	Development of related companies		331 (14.30)		Public discussions about companies that make advancements in medical AI		“Alibaba signed contracts with the First Affiliated Hospital and began to march toward AI medical treatment.”	
	Investment and market		186 (8.03)		Public discussions on the financial issues associated with medical AI		“Pony Ma, Tencent's boss, will invest in artificial intelligence medicine”	
	Industry expectations		142 (6.13)		Public prospects for the developmental trend of AI in medical care		“I think the AI medical industry has a bright future”	
	Policy and law		47 (2.03)		Public discussions on privacy and legal issues in AI industry development		“The development of AI can greatly shorten the training cycle of doctors, but the relevant legal and technical norms still need improvement.”	
**Impact on society**				622 (26.87)		Influence of AI in medical care on society		N/A
	Impact on doctors		422 (18.23)		Public perception of medical AI’s impact on human doctors		“After the development of artificial intelligence, doctors are one of the first unemployed professions. Don't let children study medicine”	
	Impact on hospitals		125 (5.40)		Public perception of medical AI’s impact on hospitals		“Will AI and telemedicine help hospitals control medical costs?”	
	Influence on public life		75 (3.24)		Public perception of medical AI’s impact on public life		“When AI enters the medical field, can human beings live forever?”	

^a^AI: artificial intelligence.

^b^N/A: not applicable.

The third category, “Impact on society” (n=622, 26.87%), includes the subcategories of impact on doctors (n=422, 18.23%), impact on hospitals (n=125, 5.40%), and influence on public life (n=75, 3.24%). Notably, people cared a lot about the impact on doctors, discussing both positive and negative implications, such as reduced workload and improved efficiency, as well as the need to deal with the threat of doctors being replaced. Medical AI would simultaneously impact health care modalities in hospitals and public life as well.

### Public Attitudes Toward AI in Medical Care

#### Overview

A total of 956 posts displayed users’ specific attitudes toward AI in medical care. Of these, 568 posts (59.4%) expressed positive attitudes, 329 (34.4%) conveyed neutral attitudes, and 59 (6.2%) highlighted negative attitudes. In order to understand why people hold these attitudes, we further analyzed the specific viewpoints in these posts (Table 3).

**Table 3:** Distributions of public attitudes toward artificial intelligence in medical care (N=956).

Attitudes		Number of posts, n (%)
**Positive attitudes**		568 (59.4)
	Artificial intelligence’s technical advantages in medical care	251 (26.3)
	Optimism about industrial development	229 (24.0)
	Helping human doctors	46 (4.8)
	Avoiding doctor-patient conflicts	24 (2.5)
	Promoting reform of medical care	11 (1.2)
	New health expectations	7 (0.7)
**Neutral attitudes**		329 (34.4)
	Information sharing, noncommittal	216 (22.6)
	Hesitation	85 (8.9)
	Commentary on both positive and negative aspects	28 (2.9)
**Negative attitudes**		59 (6.2)
	Immaturity of artificial intelligence technology	27 (2.8)
	Distrust of artificial intelligence companies	15 (1.6)
	Fear of artificial intelligence technology	7 (0.7)
	Lack of “enthusiasm” expressed by artificial intelligence	5 (0.5)
	Privacy	3 (0.3)
	Ethics and law	2 (0.2)

#### Positive Attitudes

As illustrated in Table 3, AI’s technical advantages in medical care was the most mentioned reason (251/568, 44.2%) for positive attitudes, in which people identified benefits such as high diagnostic accuracy and computational efficiency. Another main reason for positive attitudes was the optimism about industrial development (n=229, 40.3%), in which people expressed confidence and expectations for medical AI’s industrial development. Other reasons associated with AI making contributions to the medical field were also discussed, such as helping human doctors (n=46, 8.1%), avoiding doctor-patient conflicts (n=24, 4.2%), and promoting the reform of medical care (n=11, 1.9%). People believed that medical AI can not only improve human doctors’ work quality and reduce their workload but also help avoid doctor-patient conflicts. They also believed that medical AI could maintain fairness, eliminate discrimination against patients, and would not involve under-the-table fees, thus helping reduce arguments and conflicts. In addition, people felt that medical AI would affect the development of the medical system and promote the reform of medical care, which would, in turn, boost health care efficiency and benefit more patients.

#### Neutral Attitudes

Posts expressing neutral attitudes conveyed noncommittal information (216/329, 65.7%). Users mainly forwarded AI-related posts or news, either summarizing the content or including ambiguous attitudes toward medical AI (eg, “AI new technology - Nature magazine reports: AI is better than doctors in early diagnosis of autism in children”). People conveyed a sense of hesitation (n=85, 25.8%) to accept medical AI, considering that its use is not widespread and it requires time to be tested. Others provided commentary on both positive and negative aspects (n=28, 8.5%) of medical AI and did not express a preference for either view (eg, “AI may eradicate disease and poverty, but it may also destroy human beings”).

#### Negative Attitudes

For posts displaying negative attitudes toward AI in medical care, the immaturity of AI technology (27/59, 45.8%) was the leading reason for doubt. People believe AI is far from mature and must overcome many technical difficulties, including those related to obtaining high-quality medical data, such as data fraud and obstruction of hospitals (eg, “Healthcare involves huge vested interests…huge resistance needs to be overcome”), and the difficulty of standardizing medical treatment for AI (eg, “This kind of complex inspection is difficult to standardize. And it is too difficult for AI”). Distrust of AI companies (n=15, 25.4%) also accounted for negative attitudes. Some people believed that AI-related companies would use medical AI to earn money regardless of the patients’ health (eg, “AI recommends incompetent hospitals that only spend their money on advertising”). Some people mentioned a fear of AI technology (7/59, 11.9%) and the lack of “enthusiasm” expressed by AI (n=5, 8.5%), which indicated that they tended to compare AI with humans and were greatly concerned with humanistic problems. Notably, only a few people mentioned fears about the problem of privacy, ethics, and law (n=2, 3.4%) in using medical AI.

### Public Attitudes Toward Replacing Human Doctors With AI

For many decades, the fear that AI will cause widespread unemployment has waxed and waned; however, some researchers believe that fears of robots and computers greatly increasing secular unemployment are unwarranted [32]. However, the relationship between AI and human workers, especially the concern that AI workers can replace human workers, has become one of the most controversial issues in the development of the AI industry. Since this is also true in the field of medical AI, we paid special attention to public attitudes toward the issue of whether AI doctors can replace human doctors.

A total of 200 posts referred to the topic of AI replacing human doctors, and 80.0% of the posts conveyed the sense that AI doctors can completely or partially replace human doctors. Of these, 95 posts (47.5%) expressed the users’ belief that AI would replace all human doctors. We further identified the reasons for such attitudes. The results indicated that the most important reasons for people believing that AI will replace human doctors is that AI has technical advantages in medical care, such as high accuracy, stability, and efficiency. In addition, people expressed their hope that AI doctors would have the advantage of not having conflicts with their patients. There were 65 posts (32.5%) that expressed the users’ belief that AI would partially replace human doctors. Such users believed that some medical jobs, such as pathological diagnosis, are suited to AI, while others are more suitable for human doctors. To determine which kinds of doctors were considered most likely to be replaced by AI, we further coded the posts that explicitly mentioned the doctors that users believed might be completely or partially replaced by AI. Pathologists were the most frequently mentioned (n=17, 43.6%), followed by radiologists (n=8, 20.5%) and dermatologists (n=3, 7.7%). In terms of medicine and surgery, physicians (n=4, 10.3%) were considered more likely to be replaced by AI than surgeons (n=2, 5.1%).

Moreover, 40 posts (20.0%) expressed the attitude that AI will not replace human doctors. The reasons for the public holding this attitude can be classified mainly in terms of technical and humanistic concerns. First, owing to the immaturity of AI technology, people believe that it cannot adequately manage medical problems. Second, people believed patients need humanistic interactions, which AI doctors could never offer. Third, considering ethics and laws, people believed that AI doctors might invade personal privacy and lack legal supervision. Finally, a very small percentage of users opposed AI doctors based on their fear of AI technology.

## Discussion

### Principal Results

Applying AI to medical care is believed to be a promising solution for the global shortage of medical resources. As a new field, whether the medical AI industry can develop in a healthy and smooth way depends not only on numerous technological challenges, but also on whether the public can accept and trust it. However, we still lack sufficient empirical evidence to reveal actual public perception of medical AI. Based on social media data from Sina Weibo, this study took the lead in revealing the public perception of medical AI through content analysis. We mainly explored the general characteristics of people’s attention toward medical AI, the topics of public interest in medical AI, people’s attitudes toward medical AI, and their opinions on the debate regarding whether AI doctors can replace human doctors.

Overall, we found that public attention toward AI in medical care showed a noticeable event-driven trend on social media. Big social events, especially social entertainment events related to AI (eg, the world Go champion matchup between Sedol Lee and AlphaGo), generally cause a significant increase in public discussions about AI in medical care. To this end, we suggest that practitioners pay close attention to popular events related to AI and use these critical time points to promote the dissemination of knowledge related to medical AI. In addition, we revealed the profile of social media users who discussed AI in medical care; older adults, males, and people living in more affluent regions paid more attention to the issue of medical AI. These results are in accordance with previous findings that men prefer new technology products more than women [33]. As for the differences in interest across age groups, we think it may be related to the fact that older people may pay more attention to health and medical issues than younger people. Moreover, people in affluent regions may be more likely to seek information about medical AI [34] and have access to it, thus they pay more attention to it. The differential interest in medical AI between richer and relatively poorer regions in this study may support the theory of digital divide [35], and deepen the digital divide related to medical AI. How to promote the new medical technology in remote areas is also worthy of attention and future in-depth study by theorists and practitioners.

When investigating which topics interest people when discussing AI in medical care, we found that public interest mainly focused on technology and application, industry development, and societal impact. The public was most interested in AI technology and application. The specific uses of medical AI, especially in diagnosis, gained the most public attention under the category of technology and application. Our results may provide more precise guidance for the promotion and popularization of the technology and use of medical AI. In addition, the industry development and investment possibilities of medical AI (eg, development of related companies) attracted nearly one-third of public attention, among which only about 2% of posts focused on policy and law in the medical AI industry. We believe that the public's interest in industrial development is beneficial for companies that wish to rapidly promote medical AI, but the government and other regulatory authorities may need to pay attention to strengthening the formation and dissemination of relevant policies and laws in the future.

In terms of attitudes toward medical AI, our results showed that the public was optimistic, with nearly 60% of posts expressing positive attitudes. The preponderance of medical AI supporters among the general public implies that the promotion and popularization of medical AI had a relatively good public psychological basis. However, nearly 40% of public posts were neutral or opposed to AI. We found that approximately one-third of all users played the role of bystanders regarding AI in medical care. This means that the current public attitude toward AI in medicine has considerable plasticity, and neutral people may require the most attention. Moreover, some people clearly expressed negative attitudes toward medical AI. The results showed that the immaturity of AI technology, distrust of AI companies, and fear of AI were the top three reasons for these negative attitudes. On the technical side, people questioned the ability of AI and believed that it is difficult to standardize medical care, and some even expressed a natural fear regarding the safety of AI technology. This means that it will take more time for medical AI to gain people’s trust. At the organizational level, an attitude analysis revealed that people cared a lot about medical AI–related companies, and their negative attitudes could be due to their distrust of related companies (as observed with the Cambridge Analytica scandal and the #DeleteFacebook campaign). This indicates that managers from medical AI companies should not confine themselves to publicizing their technical advantages in the field of AI; rather, they should pay attention to how to actively portray a corporate image with a sense of social responsibility and trust to the public. The absence of humane care in AI was also an important reason for people’s negative attitudes, which is consistent with previous studies [36]. It is noteworthy that few posts (0.5%) were concerned about issues relating to privacy, ethics, or the law in the context of medical AI. As shown in prior studies, a collectivist culture results in a lower level of privacy concerns than in an individualistic culture [37,38]. We speculate that China’s collectivist culture and limited medical resources may have resulted in people from China being more interested about the benefits of AI than personal privacy.

On the controversial issue of whether AI could replace human doctors, we found that 80% of the posts indicated that AI doctors could replace human doctors completely or partially. This result was contrary to previous findings, which showed that 83% of medical students, most general practitioners, and the majority of physicians believe that AI will not replace them [16-18]. We speculate that this may be related to the psychological mechanism of self-defense of medical practitioners as stakeholders, which needs more direct evidence support from future research. It seems that the general public held a more open view about replacing doctors with AI than medical professionals, although the public is more conservative than AI experts. There is similar evidence that the general public was more enthusiastic about new medical technology than professional doctors [39]. Additionally, previous research also indicated that AI experts have made comments to the media that AI would soon replace doctors [15], and the public’s attitude may be affected by these comments. However, one-fifth of the posts were against AI replacing human doctors. Their pessimism about AI’s replacement of human doctors was mainly due to the immaturity of AI technology and AI’s inability to express empathy and compassion. These findings correspond with those from previous studies that showed that the role of humanistic care in medical health has become increasingly prominent [40]. It is also worth noting that the public’s attitudes on whether AI will replace human doctors may also affect medical enrollment and talent supply. Young people may be reluctant to go to medical school due to the fear that doctors will lose their jobs in the future. How to balance the potential conflict between the development of medical AI and the supply of medical talent merits discussion among governments, universities, and relevant practitioners.

### Limitations and Future Directions

While our study contributes to understanding the public perception of AI in medical care, there are a few limitations worth noting. First, as our study focused on users of the social media platform Sina Weibo, our sample may have been younger and more educated than the broader population. Since younger and more educated people may be more open to new technologies, we may have overestimated the public's optimistic attitude toward AI in medical care. Second, the data used in this study were specific to China; the unique cultural environment may have affected people’s perception of and attitudes toward medical AI; thus, the findings may not be generalizable to other countries. In this study, we found that the public in China paid much more attention to the technology and application of medical AI than to privacy risks. Whether these conclusions can be generalized to Western countries remains to be examined in future studies. However, as the most populous country in the world, the shortage of medical resources in China is severe. It is of great practical significance to explore medical AI in China. At the same time, China has the largest number of internet users in the world and has a particular advantage in the development of AI technology. Therefore, we believe that it is an important issue to clarify the Chinese public perception of and attitude toward medical AI. Nevertheless, future research can consider exploring the public perception of medical AI in other countries. Third, our analysis based on social media data has some inevitable limitations in inferring users’ apparent attitude and behavior intention. In the future, such research can be supplemented and improved with research evidence, such as a questionnaire survey.

### Conclusions

Our study presents a social listening method of assessing public perception and opinions on AI in medical care through social media content analysis. Our findings indicate that social events can easily drive the public's attention to medical AI, and that older adults, males, and people living in more affluent regions were more interested in it. The general public was most interested in AI technology and application, especially its specific uses in diagnosis, but showed little interest in policy and law in the medical AI industry. The characteristics of the public attention on medical AI that we found in this study may provide practical guidance for promoting this new medical technology. Still, we need to pay close attention to the present digital divide that may deepen further. In terms of attitudes toward medical AI, the majority of people held positive attitudes and believed that AI doctors would completely or partially replace human doctors. In the aggregate, the general public attitudes toward medical AI were more open than those of medical professionals but more conservative than those of AI experts.

Although they were generally optimistic about AI in medical care, some people still had negative attitudes toward medical AI owing to the immaturity of AI technology and their distrust of AI companies. Our results revealed that distrust of companies accounted for one-quarter of all negative attitudes toward AI. Improving the public's trust in AI companies may take more time than upgrading the technology. In addition, technical and humanistic concerns were the most important reasons for some people’s pessimism about AI replacing human doctors. We suggest that in the future, practitioners should pay more attention to humanistic care and try to meet patients’ emotional needs, rather than only focusing on technical issues. It is worth noting that currently, AI in medical care is still in its early stages, and most people have not come into contact with it. Therefore, their attitudes are likely to change with the development of medical AI products. Future research should keep track of the changes and progress of public opinion toward medical AI in the long run. We hope this study can serve as a catalyst for the understanding of public perception on medical AI and expand the ongoing conversation to additional communities. 

## Abbreviations

AI: artificial intelligence
